# Thiacalix[4]arenes Remove the Inhibitory Effects of Zn Cations on the Myosin ATPase Activity

**DOI:** 10.1186/s11671-018-2630-2

**Published:** 2018-07-25

**Authors:** Raisa Labyntsevа, Viktoriia Yavorovska, Olexander Bevza, Andriy Drapaylo, Vitaly Kalchenko, Sergiy Kosterin

**Affiliations:** 1 Palladin Institute of Biochemistry, National Academy of Sciences of Ukraine, Kyiv, Ukraine; 2 Institute of Organic Chemistry, National Academy of Sciences of Ukraine, Kyiv, Ukraine

**Keywords:** Myosin S1, Zn, Thiacalix[4]arenes, ATPase activity, Molecular docking, Smooth muscle, Uterus

## Abstract

Numerous female reproductive abnormalities are caused by uterine smooth muscle (myometrium) disorders. Heavy metals have an adverse effect on the contractility of the uterine smooth muscle. Although zinc is an essential biogenic element for most of the organisms, high doses of this element are toxic. The study of 0.5−5 mM Zn^2+^ effect on myosin S1 ATPase activity from the uterus found that 5 mM Zn^2+^ cations have the most pronounced inhibitory effect. The calculation of the kinetic parameters (*K*_m_ and *V*_max_, _ATP_) revealed that the apparent maximum velocity of the hydrolysis ATP catalyzed by myosin in the presence of 5 mM Zn^2+^ decreased by 1.6 times. The value of *К*_m_ for ATP hydrolysis by myosin S1 in the presence of Zn^2+^ does not change statistically, although it tends to decrease. It was determined that uterine myosin S1 ATPase activity does not depend on the concentration of Mg^2+^ in the presence of 5 mM Zn^2+^. Also, it was demonstrated that tetrahydroxythiacalix[4]arene-tetrasulfosphonate (C-798) and tetrahydroxythiacalix[4]arene-tetraphosphonate (C-800) restored myosin S1 ATPase activity to the control level in the presence of 5 mM Zn^2+^. One of the most probable mechanisms of restoring the action of these thiacalix[4]arenes protective effect is based on its ability to chelate heavy metal cations from the incubation medium. The molecular docking of C-798 and C-800 into the myosin S1 region showed that these thiacalix[4]arenes could interact with Zn cation bond by myosin amino acid residues near the ATPase active site. Therefore, thiacalix[4]arenes may weaken the interaction between this cation and myosin S1. It was speculated that the obtained results could be used for further research with the aim of using this thiacalix[4]arenes as pharmacological compounds in the case of poisoning with high concentrations of zinc.

## Background

The problem of pollution of the environment with heavy metals and the search for ways to reduce their impact on living objects is relevant [1, 2].

Zinc is an essential biogenic element for most of the organisms. [3]. Zinc ions form complexes with multiple proteins that carry out vital metabolic functions. The zinc ion is a component of at least 300 metalloenzymes that catalyze more than 50 different biochemical (physiological) reactions [4, 5]*.*

However, zinc is a heavy metal. It can be found in group IIb of the periodic table of the elements, together with the two toxic metals cadmium and mercury. Nevertheless, zinc is considered to be relatively non-toxic to humans [6]. This element is noxious only in excessive doses [7].

The oral LD_50_ for zinc is close to 3 g/kg body weight according to the TOXNET database of the US National Library of Medicine. It is more than 10-fold higher than cadmium and 50-fold higher than mercury [6]. The excess of the normal concentration of this microelement in humans is most commonly caused by the intake of medications and biologically active additives containing redundant zinc in their composition. It was recorded as individual cases of intoxication with zinc as a result of eating food stored in zinc-coated or fully zinc containers. Zinc oxide, chloride, and zinc sulfate are *extensively used in the industry* for the production of glass; in the manufacture of artificial fibers, zinc paints, ceramics, matches, and dental cement; in the pulp and paper industry, for preserving wood, and for tinning and soldering.

A high concentration of zinc intake altered the immune response [8]. The elevated levels of Zn, Al, Cu, and Fe in the brain may facilitate the development or progression of Alzheimer’s disease according to some epidemiological studies [9, 10].

Heavy metals can affect the female reproduction on different stages such as the beginning of fetal life, early development, and maturation. Cations of heavy metals also can be the cause of subfertility, infertility, intrauterine growth retardation, spontaneous abortions, malformations, birth defects, postnatal death, premature aging, and learning and behavior disorders [11, 12].

The uterine contractile function is associated with the activity of the protein complex—actomyosin—in which myosin exhibits enzyme activity, namely the ability to hydrolyze ATP. Myosin ATPase localized in the catalytic domain of the subfragment-1 (S1 or head) transforms chemical energy deposited in macroergic bonds of ATP into mechanical movement. As a result, myosin moves along the actin filament, causing the muscle contraction. Therefore, ATP hydrolysis catalyzed by myosin is considered as one of the essential processes in the molecular mechanism of the myometrial function [13, 14].

The myosin subfragment-1 is an N-terminal part of the myosin heavy chain which consists of two domains: the N-terminal globular motor (catalytic) domain containing the ATP-ase site and the actin-binding site, and the regulatory domain, or lever-arm responsible for the movement of myosin along the actin filaments. The core of the myosin motor domain is formed by a central, seven-stranded β-sheet which is surrounded by α-helices. A large structural domain that accounts for six of the seven strands of the central β-sheet is usually referred to as the upper 50-kDa domain (U50). A large cleft separates the upper 50-kDa domain from the well-defined structural lower 50-kDa domain (L50) which is formed by amino acid residues from 465 to 590. The actin-binding region and nucleotide-binding site of myosin are on opposite sides of the seven-stranded β-sheet with the phosphate moiety of the nucleotide at the rear of the nucleotide-binding pocket. The P-loop, switch 1, and switch 2 are located in the upper 50-kDa domain close to the apex of the large cleft. All three nucleotide-binding motifs contact with the phosphate moiety of the nucleotide at the rear of the nucleotide-binding pocket and act as γ-phosphate sensors [15].

It was found in our previous studies that heavy metal cations inhibited the myosin ATPase activity of the uterine smooth muscle [16, 17] that can negatively affect the contractile properties of the myometrium.

The adverse impact of heavy metal on the uterine contractility requires the development of pharmacological substances that can eliminate these harmful effects.

Calixarenes have currently attracted the attention of researchers as prospective artificial effectors for different biochemical processes. These compounds are synthetic macrocyclic phenol oligomers which have a cup-shaped structure. Their upper and lower rims can be functionalized with various chemical substituents. Calix[4]arenes are formed by four functionalized arene fragments and characterized by a rather flexible macrocycle conformation. Calix[4]arenes showed low toxicity of the matrix and the ability to penetrate into the cells. Hence, these compounds are regarded as promising agents for developing new effective drugs [18, 19].

A promising class of such substances is water-soluble thiacalixarenes [18] possessing the metal complexing groups at the macrocyclic molecular platform. The calixarenes due to their ability to form the supramolecular complexes with (bio)metal cations have also been used in biomedical research as extractants of heavy metals [20–22].

We have shown previously that tetrahydroxy-thiacalix[4]arene-tetrasulfonate (С-798) eliminated the inhibitory effects of Pb^2+^, Cd^2+^, and Ni^2+^ on ATP hydrolysis catalyzed by myosin S1 from swine myometrium [23].

This study aimed to research the effect of high concentrations of zinc cations and their joint action with tetrahydroxy-thiacalix[4]arene-tetrasulfonate (С-798) and tetrahydroxy-thiacalix[4]arene-tetraphosphonate (C-800) on the myosin S1 ATPase activity from the uterus. This study was needed to test the ability of these thiacalixarenes to eliminate the adverse effects of high concentrations of zinc on the enzymatic activity of the uterine myosin.

**Fig. 1:**
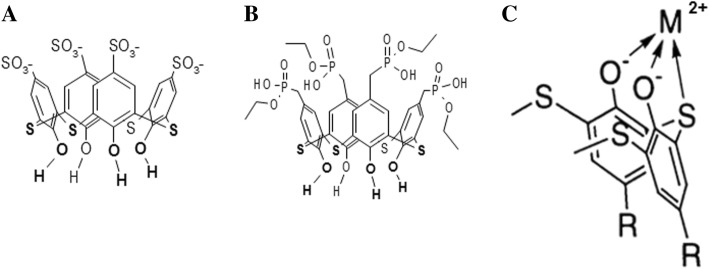
The chemical structure of the tetrahydroxy-thiacalix[4]arene-tetrasulfonate (C-798) (**a**), tetrahydroxy-thiacalix[4]arene-tetraphosphonate (C-800) (**b**), and the scheme of the chelating complex of the thiacalixarene with metal cation on the lower rim (inverted position) (**c**)

Thiacalix[4]arenes C-798 and C-800 consist of a cup formed by four phenolic fragments modified at the upper rim with four anionic sulfonate and four phosphonate groups, respectively. Both thiacalix[4]arenes have hydroxyl groups and bivalent sulfur atoms densely located on the lower rim allowing them to chelate heavy metals with the formation of stable metal complexes [21] (Fig. 1).

This work is the result of a joint project Palladin Institute of Biochemistry and Institute of Organic Chemistry of NAS of Ukraine focused on the interaction of myosin ATPase of myometrium with calix[4]arenes that are inhibitors or activators (effectors) of uterine myosin ATPase.

## Results

### Myosin S1 ATPase Activity Dependence on Zn^2+^ Concentration

**Fig. 2:**
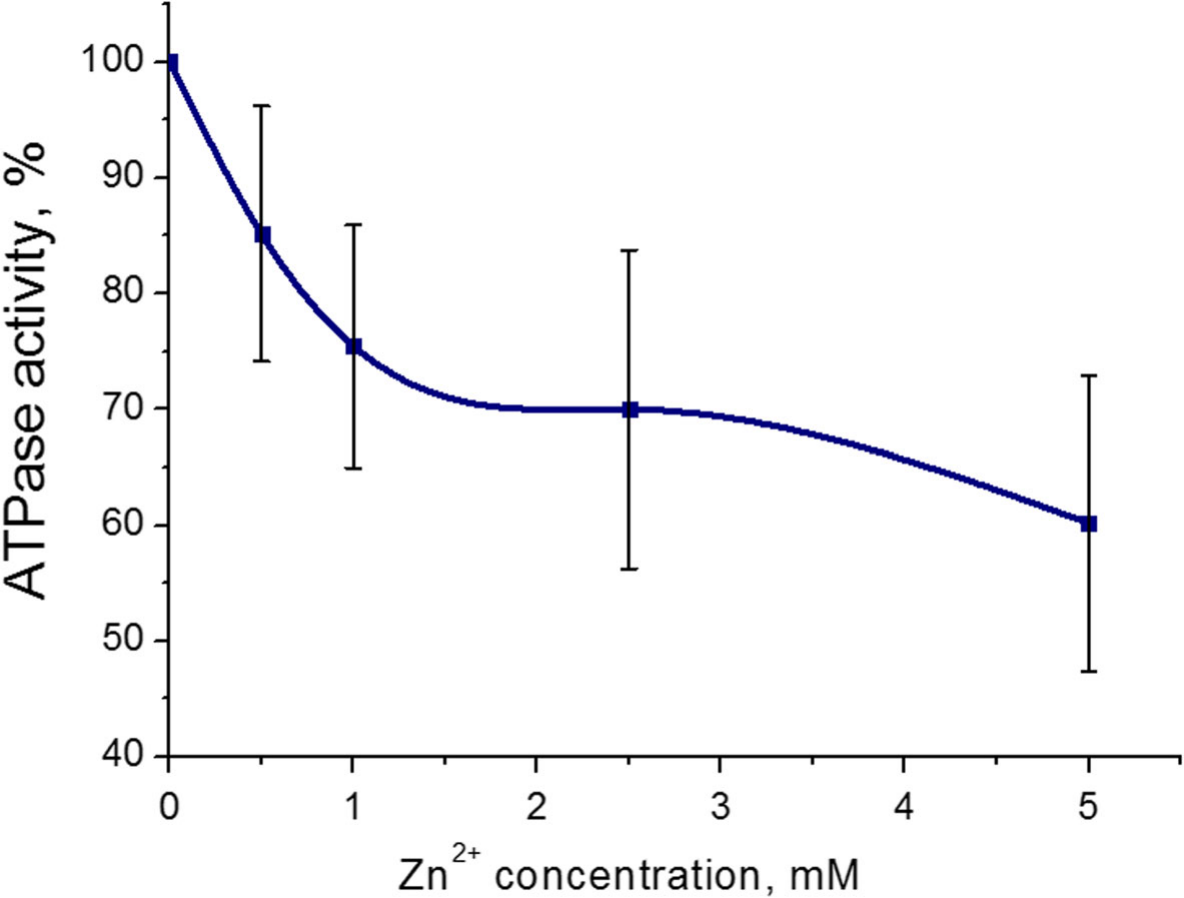
Myosin S1 ATPase activity from the myometrium in the presence of 0.5–5.0 mM concentrations of Zn cations (M ± SD, *n* = 6). 100% is the value of ATPase activity without the addition of Zn cations

It was found that most pronounced inhibitory effect on myosin S1 ATPase activity from the uterus was at 5 mM (43 ± 8%, M ± SD) for Zn cations. The concentration range of Zn^2+^ was 0.5−5 mM in the incubation medium (containing 3 mM ATP, 5 mM Mg^2+^, and 0.01 mM Ca^2+^). One hundred percent is the value of ATPase activity without the addition of Zn cations (control) (Fig. 2). Thus, the adverse effects of Zn cations on the myosin S1 ATP hydrolysis were further studied with 5 mM of Zn^2+^.

### Myosin S1 ATPase Activity Dependence on ATP Concentration in Presence 5 mM Zn^2+^

**Fig. 3:**
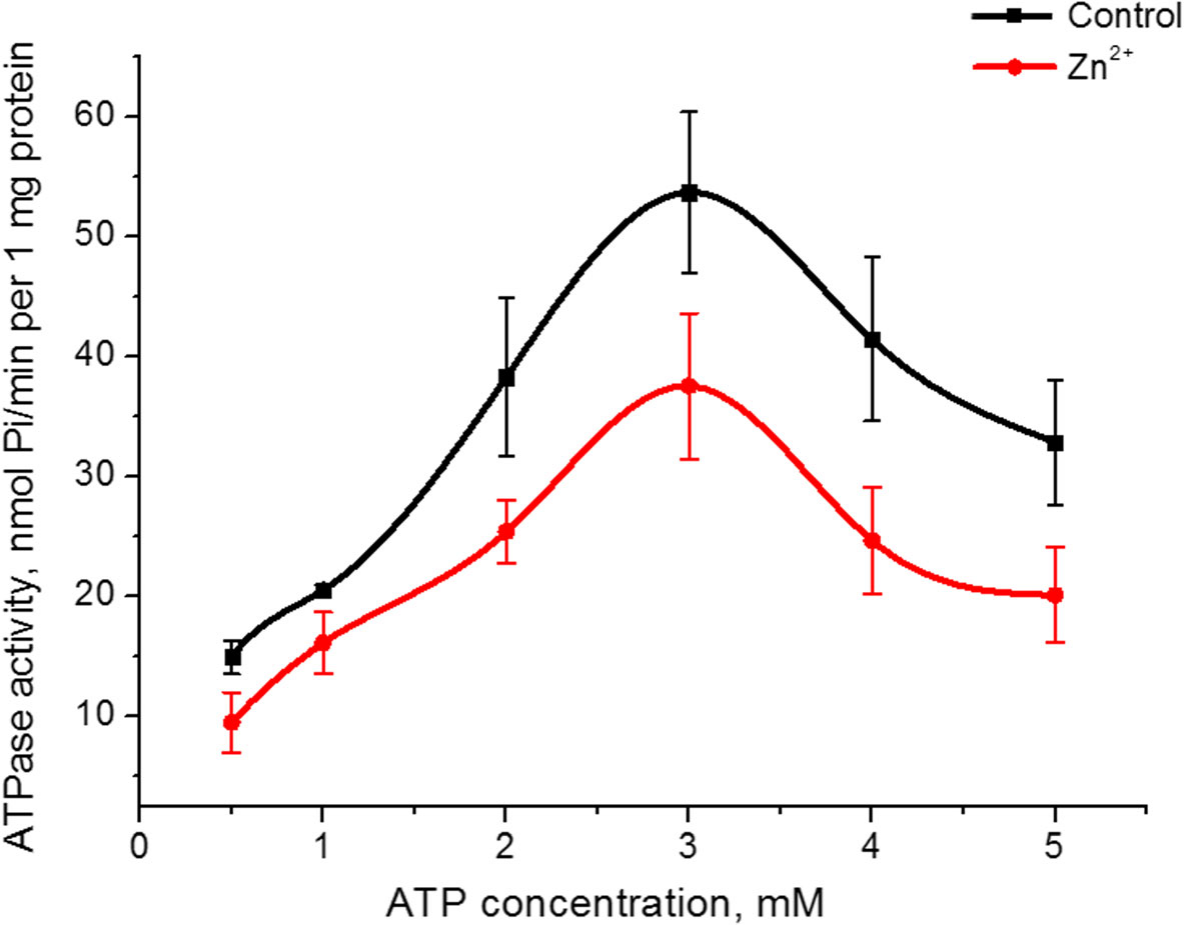
The influence of 0.5–5 mM ATP on myosin S1 ATPase activity from the uterus in the presence of 5 mM Zn^2+^ in comparison with a control (M ± SD, *n* = 5)

The Zn^2+^ effect on the affinity of the myosin S1 ATPase activity to its substrate (ATP) was investgated. Increasing the ATP concentration in the incubation medium from 0.5 to 5 mM at a fixed MgCl_2_ concentration (5 mM) both in the control and in the presence of 5 mM Zn^2+^ resulted in a dome-shaped graph with a maximum value of ATPase activity at 3 mM ATP. The value of enzymatic activity at this peak in the presence of zinc was 30% lower than that of the control (Fig. 3). The graphs of the dependence of the myosin S1 ATPase activity on the ATP concentration in the control and the presence the 5 mM Zn^2+^ in the ascending part were linearized according to Lineweaver–Burk method [27]. The calculation of the kinetic parameters namely the imaginary constant of Michaelis (*K*_m_) and maximal rate of myosin S1 ATPase for ATP (*V*_max, ATP_) revealed that *V*_max, ATP_ of the myosin enzymatic activity in the presence of 5 mM Zn^2+^ decreased by 1.6 times (38 ± 7 and 22 ± 6 nmol Pi/min per 1 mg protein in the control and the presence of Zn^2+^ respectively, *n* = 5). The value of *К*_m_ for ATP hydrolysis by myosin S1 does not change statistically, although it tends to decrease (0.49 ± 0.15 mM in the control, 0.38 ± 0.12 mM in the presence of Zn^2+^; M ± SD; *n* = 5).

### Myosin S1 ATPase Activity Dependence on Mg^2+^ Concentration in Presence 5 mM Zn^2+^

**Fig. 4:**
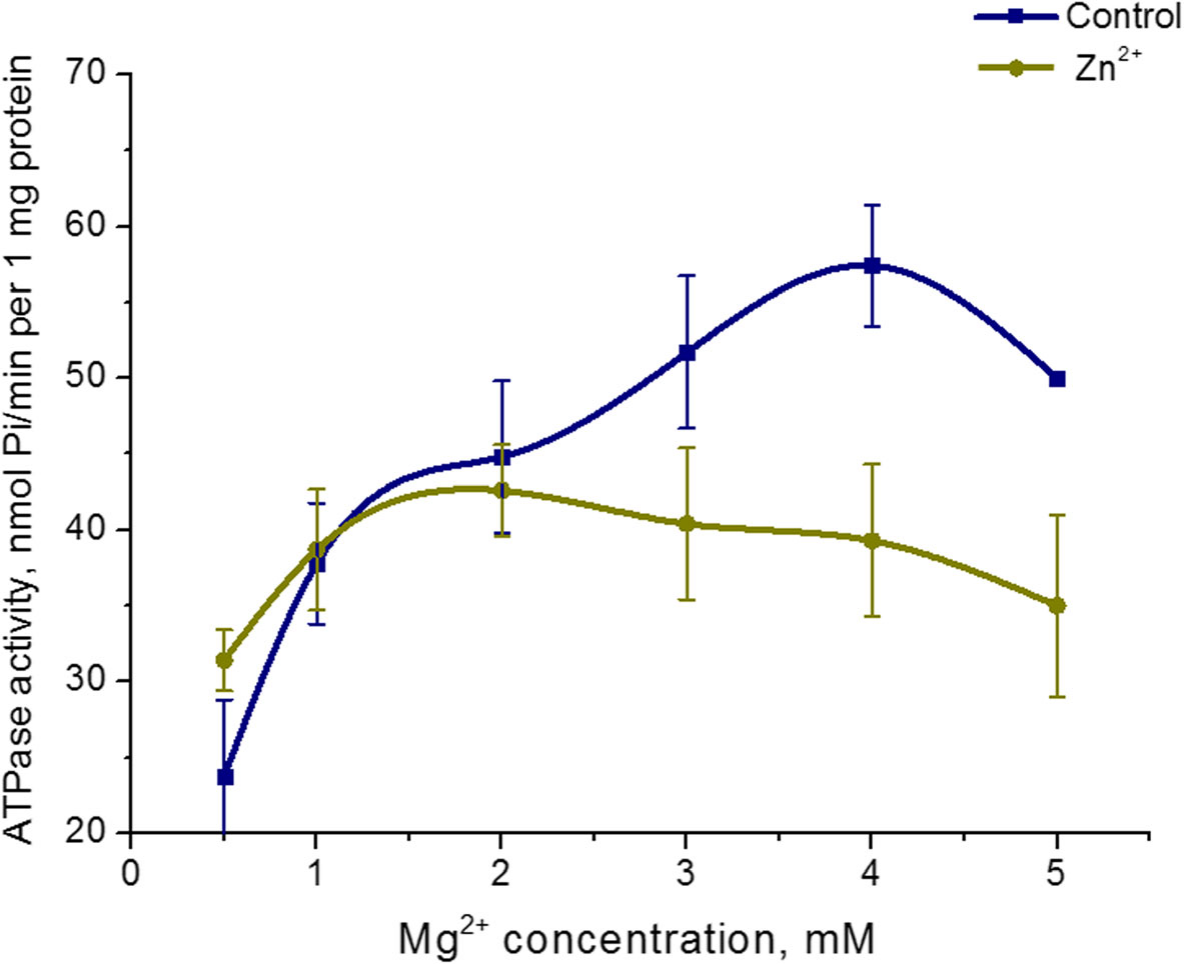
Myosin S1 ATPase activity dependence on Mg^2+^ concentration in the presence of 5 mM Zn^2+^ in comparison with a control (M ± SD, *n* = 6)

The effect of 5 mM Zn^2+^ on Mg^2+^ concentration dependence on ATPase activity of uterine myosin was studied. Increasing the Mg^2+^ concentration in the incubation medium from 0.5 to 5 mM at a fixed ATP concentration (3 mM) in the presence 5 mM Zn^2+^ does not result in a change of myosin S1 ATPase activity. At the same time, the Mg^2+^ concentration dependence of ATPase activity in the control (in the standard conditions) was detected. The highest level of myosin ATP hydrolysis in the control was achieved at 3 mM Mg^2+^ (Fig. 4). Therefore, the myosin S1 enzyme activity of the uterus does not depend on the concentration of Mg^2+^ in the presence of Zn high concentrations (5 mM).

### Zn-Binding Sites in Myosin S1

The computer simulation shows that Zn cations have several binding regions in myosin head. One of them is located in the lower part of the cleft between the upper and lower 50-kDa subdomains, close to the nucleotide-binding site, and directly near the P-loop. Zn^2+^ is coordinated with the oxygen atoms Glu177 (bond length 0.23 and 0.39 nm), with the Ser178 oxygen atom (bond length 0.31 nm), and Arg236 (bond length 0.32 nm).

Other Zn-binding region is located at the bottom of the upper 50-kDa subdomain (Leu218-Asp463, Glu605-Phe621) and close to the switch 1 (Gly233-Phe246) and P-loop. Zn^2+^ can coordinate with the Glu327 oxygen atom (bond length 0.21 nm), with an oxygen atom Glu326 (bond length of 0.34 nm) and an oxygen atom Asp323 (bond length 0.32 nm). The Zn cation may also interact with the myosin S1 in the region that contacts the switch 2, interacting with Glu 465 (0.24 nm), Asp468 (bond length 0.31 nm), and Leu653 (bond length 0.37 nm). This binding region is near the actin-binding site and the cleft between the upper and lower 50 kDa subdomains. The bottom of this cleft is located in the ATP-binding pocket. These binding Zn^2+^ myosin S1 domains play an essential role in the binding and hydrolysis of the ATP. These regions undergo complex conformational transformations in the process of transferring energy from the ATP-hydrolysis site to the actin-binding surface.

### Thiacalix[4]arenes Eliminate the Inhibitory Effects of Zn^2+^ on the Myosin ATPase Activity

**Fig. 5:**
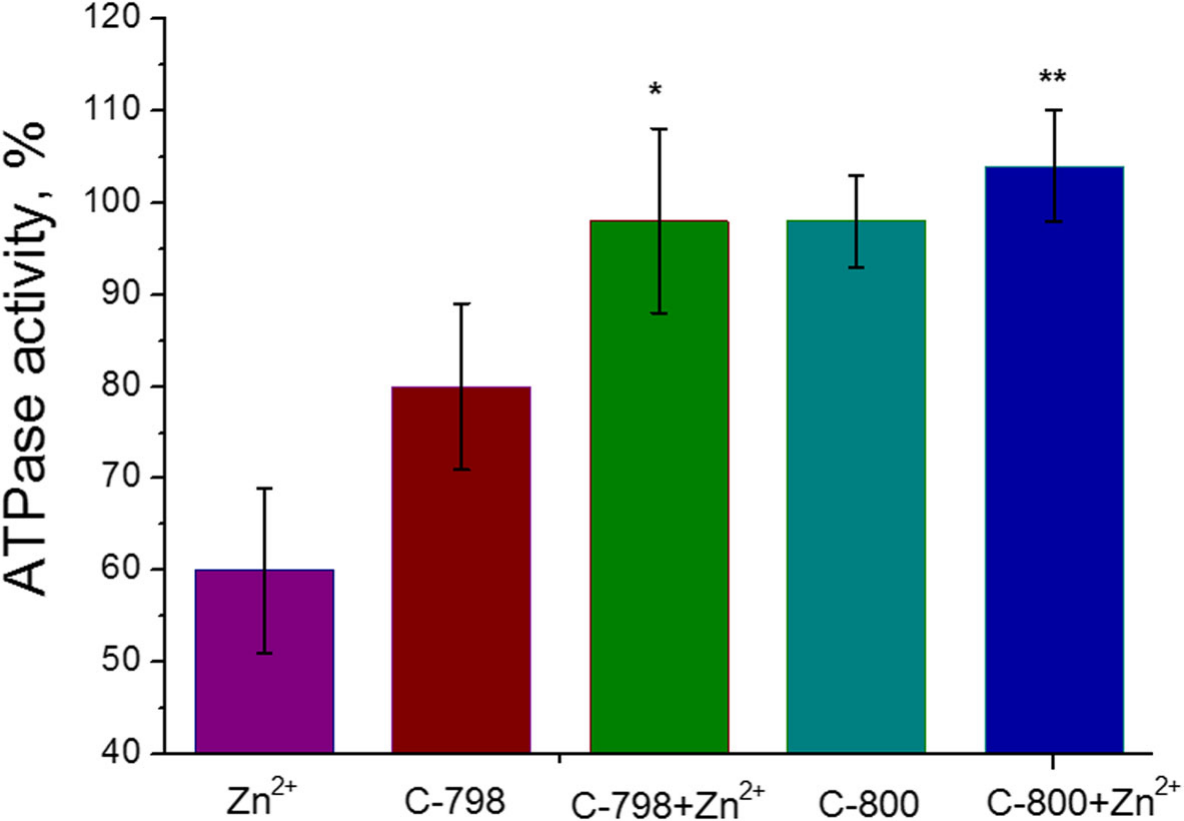
Effect of 100 μM C-798 and C-800 on myosin S1 ATPase activity in the presence of 5 mM Zn^2+^ (M ± SD, *n* = 5–6). 100% is the value of ATPase activity without the addition of Zn cation. The difference between the “Zn” and “Zn + C-798,” as well as between the values of “Zn” and “Zn + C-800,” is statistically significant (*p* < 0.05) and shown as * and **, respectively

One hundred micromolars of C-800 or C-798 solutions in 50 mM Tris-HCl buffer (pH 7.2) was added to the incubation medium containing 5 mM Zn cations to remove the negative impact of Zn^2+^ on the ATPase activity of the uterine smooth muscle myosin S1. As a control, it was used as enzymatic activity without adding zinc and/or thiacalix[4]arenes to the incubation medium. It was shown (Fig. 5) that compound C-800 does not affect the ATPase activity of myosin S1 of the myometrium. Although, compound C-798 exhibits a small inhibitory effect on the myosin S1 ATPase activity that is most likely related to the extraction of a certain amount of Mg^2+^ [21], essential for ATP binding in the active center and its hydrolysis, from the incubation medium. Nonetheless, 100 μM C-798, as well as C-800, removed the inhibitory effects of 5 mM Zn^2+^ on the process of ATP hydrolysis catalyzed by myosin S1.

### Probable Mechanisms of C-798 and C-800 Restoring Action on the Myosin S1 ATPase Activity in the Presence of the Zn^2+^

One of the most probable mechanisms of C-798 and C-800 restoring action on the myosin S1 ATPase activity in Zn^2+^ presence can be the ability of thiacalix[4]arenes to bind Zn cations and, consequently, exclude these cations from the incubation medium. It was interesting if these thiacalix[4]arenes can bind to zinc cations that are already bound to myosin.

The computer simulation showed that thiacalix[4]arenes C-798 and C-800 with bridge sulfur atoms between aromatic rings are in the conformation of “cone” stabilized by intra-hydrogen bonds between the phenolic groups. The energy-minimized structure of these calixes[4]arenes was obtained. The total energy of C-798 after the minimization was 64.5 kcal/mol. The presence of ionized groups on the calix[4]arene rims (in particular the lower) significantly increases the contribution of electrostatic interactions to the total energy of the interaction of the host-guest. We also carried out the “minimization” of the C-798—Zn^2+^ complex; its total energy was 83 kcal/mole.

**Fig. 6:**
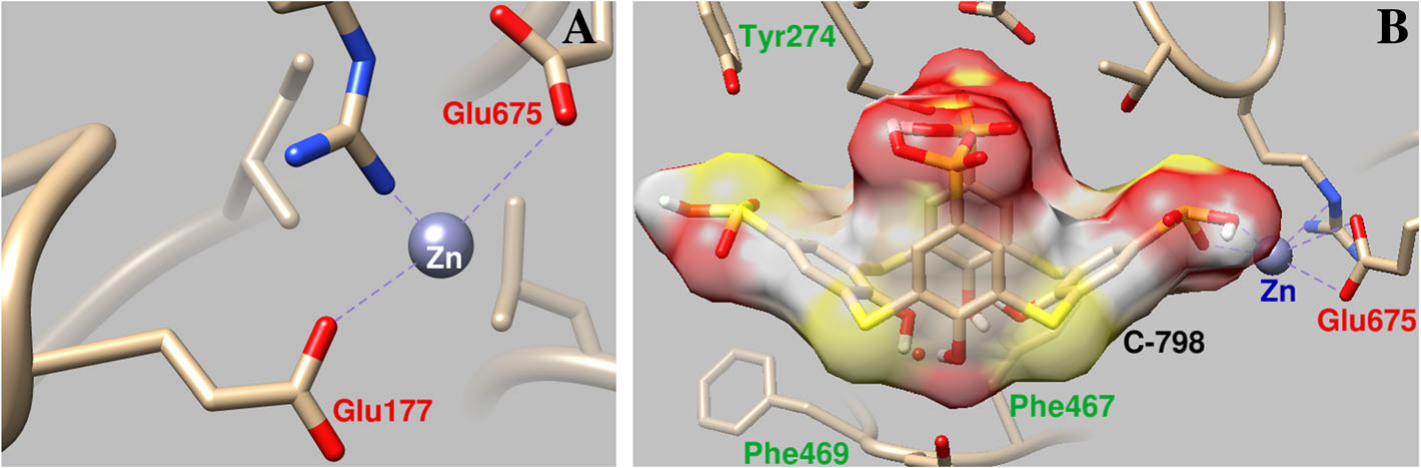
Geometric parameters of the interaction of Zn^2+^ with the P-loop region of myosin S1 (**a**) and the influence of C-798 on the cooperation of Zn cation with this region (**b**)

C-798 was embedded in the structure of the myosin S1 cooperating with Zn cation that was previously bound to the protein in the region of the loop P. In this case, Zn^2+^ interacts with the lower rim oxygen atoms and bridge sulfur of C-798 (O3, 0.21 nm; S1, 0.30 nm; O2, 0.34 nm). It is shown that Zn^2+^ deviates to some extent from the amino acid residues of the P-loop and weakens its interaction with the Glu177 oxygen atom (0.43 nm bond length) (Fig. 6).

Fixation of the C-798 in the “cavity” of the ATP-binding region of myosin occurs with the participation of several amino acid residues. In particular, the hydrophobic basket of thiacalixarene has fixed by the myosin aromatic amino acid residues of Phe467 and Phe469; thiacalixarene negatively charged oxygen atoms interact with positively charged amino acid residues of Arg570, Asn572, and His689.

**Fig. 7:**
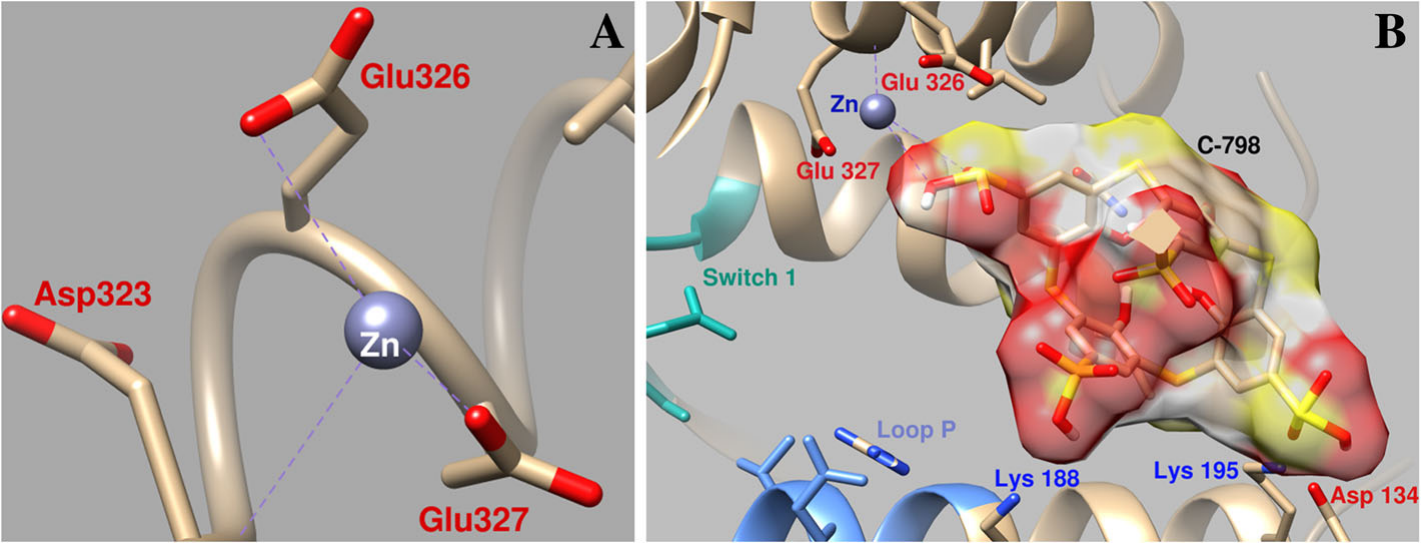
Geometric parameters of the interaction of Zn^2+^ in the area near the myosin switch 1 and P-loop of myosin S1 (**a**) and the influence of C-798 on the cooperation of Zn^2+^ with this region (**b**)

The study of the C-798 influence on the change of the Zn^2+^position during docking in the area near the myosin ATP-binding site demonstrated that Zn^2+^in the presence of thiacalix[4]arene interacts with the oxygen atoms of the third sulfonyl group (O16–0.26 nm; O15–0.27 nm), almost does not interact with the oxygen atoms Asp134 and Glu326, and coordination with Glu327 is much weaker (0.43 nm coupling length) (Fig. 7). In this case, thiacalixarene is fixed in the “cavity” of the protein with the participation of several amino acid residues. In particular, negatively charged oxygen atoms of thiacalixarene sulfonyl groups interact with myosin positively charged amino acid residues of Lys188, Lys195, and Gln221.

It is shown that the conditional energy sphere of Zn cation contacts and even somewhat overlaps with the surface of the spread of electrostatic interactions due to the presence of oxygen atoms and sulfur atoms in C-798. This indicates that the cation being studied is in close enough interaction with negatively charged atoms of the upper and lower C-798 crowns. It is probable that thiacalixarene pulls on itself a Zn^2+^, as a result of which the interaction of cations with the amino acid residues of the enzyme is weakened.

Consequently, the results of the C-798 docking in the myosin S1 region, which contains Zn^2+^ bound, indicate the possibility of the interaction of C-798 functional groups with Zn cation. In this case, the Zn cation bond with the amino acid residues of the myosin S1 was significantly weakened, and the distance between them increases. As a result, the adverse effect of Zn cation on the ATPase activity of myosin can be eliminated.

We also conducted a computer simulation of the effect of calix[4]arene C-800, with docking in the area close to the ATP-binding region of S1 myosin, to change the geometry of the cation Zn. At the same time, Zn^2+^ is in contact with Arg236 (0.37 nm bond length), Glu675 (0.41 nm bond length), and atoms of the upper functional calix[4]arene residues (H26, 2.36 nm; H30, 2.96 nm; C30, 0.31 nm; O5, 3.7 nm; O16, 0.4 nm; O7, 0.48nm; and O11, 0.51 nm bond length). Zn^2+^ interacts with calix[4]arene C-800, similar to C-798. The cation is also withdraw from the previous binding site in this region, and is in contact with Asp468 (0.2 nm bond length) and atoms of the lower calixarene crown (C4, 1.96 nm; C14, 2.07 nm; S3, 2.16 nm; O2, 2.26 nm; C3, 2.97 nm; C20, 3.10 nm; O4, 4.2 nm; C14, 3.0 nm; and O3, 4.1 nm).

C-798 and C-800 docking into the myosin subfragment-1 region showed that these thiacalix[4]arenes could interact with Zn cations binding to myosin amino acid residues near the ATPase active site. Therefore, their protective effect might be the result of the weakening of the interaction between these cations and myosin S1.

## Discussion

Numerous female reproductive abnormalities are caused by uterine smooth muscle (myometrium) disorders. Heavy metals have an adverse effect on the contractility of the uterine smooth muscle. Heavy metal zinc is an essential biogenic element for most of the organisms; high doses of this element are toxic [5]. Several investigations of the millimolar concentrations of Zn^2+^ on living objects were previously described [32]. We found that 5 mM Zn have the most pronounced inhibitory effect on myosin S1 ATPase activity from the uterus. Thus, the adverse effects of Zn cations on the myosin S1 ATP hydrolysis have been further studied with this concentration of Zn^2+^. The calculation of the kinetic parameters of myosin S1 ATPase for ATP revealed that *V*_max, ATP_ of the myosin enzymatic activity in the presence of 5 mM Zn^2+^ decreased by 1.6 times. The value of *К*_m_ for ATP hydrolysis does not change statistically, although it tends to decrease.

Myosin in physiological conditions is a Mg^2+^-dependent ATPase. Magnesium cation is involved in ATP binding in the myosin active site, as well as in ATP hydrolysis. The Mg^2+^ is coordinated in the active site of the enzyme with the lateral chains of the myosin amino acid residues Thr-186 and Ser-237 as well as β- and γ-phosphate groups of the ATP molecule with the formation of the β- and γ-bidentate complex as well as with active water molecules, one of which carries out a nucleophile attack on γ-phosphate ATP [33, 34]. Mg^2+^ interacts with ATP negatively charged phosphorus groups, polarizes them, and thus facilitates a nucleophilic attack on terminal γ-phosphate [14].

It was found that the myosin S1 ATPase activity is not sensitive to the presence of Mg^2+^ at 5 mM concentration of Zn^2+^ in contrast to the control when zinc was absent in the incubation medium [35, 36].

The myosin ATPase activity depends on the metal cation nature and correlates well with their ionic radius. The ion radii Mg^2+^ and Zn^2+^ in the solutions are very similar (0.070 and 0.076 nm, respectively) [37]. Therefore, the interaction of Zn^2+^ cations with the Mg^2+^-binding sites of myosin is possible. Thus, the Mg^2+^-binding sites can be occupied by Zn^2+^ cations in its high concentrations. ATPase activity of the myosin S1 in such conditions may be insensitive to magnesium cations. Myosin contains two high-affinity sites for Mg^2+^, and Mg^2+^ bound at these sites has an important physiological role in the energy transduction process during muscle contraction. There are still several Mg^2+^-binding sites in addition to the ATPase site in the myosin molecule, differing in the binding energy of magnesium ions and their affinity [35]. Therefore, it can be assumed that Zn^2+^ can also bind to other functionally important sites of myosin S1 that affect the binding and hydrolysis of ATP.

The computer simulation shows that Zn cations have several binding regions in myosin head located close to ATP binding site namely P-loop and upper and lower 50-kDa subdomains switch 2. These binding Zn^2+^ myosin S1 domains play an essential role in the binding and hydrolysis of the ATP. These regions undergo complex conformational transformations in the process of transferring energy from the ATP hydrolysis site to the actin-binding surface.

The analyze results obtained by Zn^2+^ docking into myosin S1 indicate that a key role in the binding of this cation to the myosin molecule plays its interaction with negatively charged groups of the enzyme amino acid residues, in particular, Glu and Asp.

The harmful influence of toxic concentrations of Zn^2+^ cations on the myosin S1 ATPase activity requires the search for pharmacological compounds that can eliminate the action of this metal. The objects of our study were tetrahydroxythiacalix[4]arene-tetrasulfonate (C-798) and tetrahydroxythiacalix[4]arene-tetraphosphonate (C-800) that are capable of chelating transitional and heavy metals with the formation of stable metal complexes (Fig. 1). The upper macrocyclic crown of C-798 and C-800 contains four anionic sulfonate groups or four phosphonates, respectively, which provide good water solubility of thiacalixarene and adhesion to protein molecules due to electrostatic contacts with positively charged nitrogen atoms of amino acid fragments [21].

C-798 and C-800 docking into the myosin S1 region showed that these thiacalix[4]arenes could interact with Zn cations binding to myosin amino acid residues near the ATPase active site. Therefore, their protective effect might be the result of the weakening of the interaction between these cations and myosin S1. It was speculated that the obtained results could be used for further research with the aim of using this thiacalix[4]arenes as pharmacological compounds in the case of poisoning with high concentrations of zinc.

## Conclusions

High concentration (5 mM) of Zn cation inhibited myosin S1 ATPase activity from the uterus. Inhibited influence of Zn is related by a decrease of maximal velocity of the hydrolysis ATP catalyzed by myosin S1 in the presence of 5 mM Zn^2+^. The value of *К*_m_ for ATP does not change statistically, although it tends to decrease.

Tetrahydroxythiacalix[4]arene-tetrasulfosphonate (C-798) and tetrahydroxythiacalix[4]arene-tetraphosphonate (C-800) restored myosin S1 ATPase activity to the control level in the presence of 5 mM Zn^2+^.

Zn cations have several binding regions in myosin S1 located close to the ATPase active site. The docking of C-798 and C-800 into the myosin S1 region, which contains Zn^2+^ bound, indicates the possibility of the interaction of these thiacalix[4]arene functional groups with bound Zn cations. The Zn cation bond with the amino acid residues of the myosin S1 was significantly weakened, and the distance between them increases. As a result, the adverse effect of Zn cation on the ATPase activity of myosin can be eliminated.

It is assumed that the obtained results could be used for further research with the aim of using this thiacalix[4]arenes as pharmacological compounds in the case of poisoning with high concentrations of zinc.

## Methods

### Reagents

The following reagents were used: serum albumin, EGTA, EDTA, ATP, ascorbic acid, Tris, tricine, dithiothreitol, acrylamide, (Sigma, USA), glycine (Merck, Germany), N, N′-methylenebisacrylamide (Acros Organics, Belgium) N,N,N′,N′-tetramethylenediamine (Reanal, Hungary), and reagents of domestic production (R grade). The solutions were prepared in water purified on Crystal Bio system (Adrona, Latvia). The water conductance was less than 0.1 μS. The concentration of the divalent metal cations in solution was determined by the Mohr method.

### Actomyosin and Myosin Subfragment-1 Isolation

Actomyosin was isolated from pig uterine smooth muscle by the modified Barany method as described in [17]. Myosin S1 was obtained from pig actomyosin by the modified Suzuki method [24]. The purity of the samples was controlled by PAAG-SDS electrophoresis [25].

### ATPase Activity Assay

ATPase activity of myosin S1 was determined in a 96-well plate at 37 °C in an incubation medium (total volume 0.1 ml) of the following composition (mM): Tris-HCl buffer (pH 7.2), 20; KCl, 100; CaCl_2_, 0.01; MgCl_2_, 5; and ATP, 3 (standard conditions). Protein (myosin S1) concentration was 20 μg/ml. Incubation time was 5 min. Samples containing all components of the incubation medium without myosin S1 were taken as control of non-enzyme hydrolysis of ATP. The amount of inorganic phosphate released during ATP hydrolysis reaction was determined by the Chen method [26] by the measurement of optical absorbance of the solution at 820 nm using a microplate reader μQuwant (Biotek^@^ Instruments, Inc., USA) and specified as P_i_ nmol/min per 1 mg of protein.

The Zn^2+^ and thiacalix[4]arene effects on the ATPase activity of myosin S1 were studied using standard incubation medium with solutions of ZnCl_2_ and thiacalix[4]arenes at the corresponding concentrations. The value of ATPase activity in the absence of ZnCl_2_ and/or calix[4]arenes in the incubation medium was taken as 100% (control).

### Kinetic and Statistical Analysis

The values of the imaginary constant of Michaelis (*K*_m_) and maximal rate of myosin S1 ATPase for ATP (*V*_max, ATP_) were calculated using the graph of the dependence of ATPase activity on the ATP concentration according to Lineweaver–Burk method [27]. Statistical processing of the obtained data was performed using standard methods of variation statistics. Experimental data were analyzed by using the standard software “MS Office” and “Statistica 4.5.” The statistical comparisons were performed using two-way analysis of variances (ANOVA).

### Thiacalix[4]Arene Synthesis and Characterization

Tetrahydroxy-thiacalix[4]arene-tetrasulphonate and tetrahydroxy-thiacalix[4]arene-tetraphosphonate were synthesized and characterized using NMR techniques and IR spectroscopy in the Phosphoranes Chemistry Department of the Institute of Organic Chemistry, NAS of Ukraine. Infrared and NMR spectroscopy confirmed the structure of these synthesized thiacalix[4]arenes. This thiacalix[4]arenes were dissolved in water.

### Computer Modeling

Computer modeling of the interaction between ligands (thiacalix[4]arenes, Zn^2+^, model bindings) and receptor (myosin S1) was performed using AutoDock software, version 4.2 [28]. We used the three-dimensional enzyme structure with the 1b7t identifier in RSCB PDB in our research [29]. Computer modeling of the thiacalix[4]arene structural peculiarities was carried out using HyperChem 7.01. Molecular dynamics calculations were performed by the MM2 method with the semi-empirical methods (CNDO).

Program AutoDockTools was used for preliminary “processing” of interacting molecules. One hundred runs of Lamarkian genetic algorithms (population size, 100; the maximal number of energy evaluations, 10^6^) were conducted. To analyze and visualize the docking results, we used the programs Chimera [30] and Yassara [31]. Calculation of the minimal total binding energy was implemented considering Van der Waals forces, electrostatic and hydrophobic interactions, and hydrogen bonds. The optimal ligand positions in the complex “receptor-ligand” were determined according to the energy values obtained by docking software calculator for binding energy in complex “receptor-ligand.” Thus, we selected a series of complexes with the lowest total energy and then calculated the optimal geometry of the complexes and determined the most energetically preferred arrangement of the ligands in the space of myosin subfragment-1 binding domain.

## Abbreviations

C-798: Tetrahydroxythiacalix[4]arene-tetrasulfosphonate
C-800: Tetrahydroxythiacalix[4]arene-tetraphosphonate
CNDO: Complete Neglect of Differential Overlap *(methods)*
*K*_m_: Michaelis constant, the substrate concentration at which the reaction rate of the enzyme is half of the maximal velocity
L50: Lower 50-kDa domain of myosin
LD_50_: Lethal dose is the amount of an ingested substance that kills 50% of a test sample
MM2: A class of force fields
Myosin S1: Myosin subfragment-1
NASU: National Academy of Science of Ukraine
PDB: Protein Data Bank
P-loop: Phosphate-binding loop of myosin
RCSB: Research Collaboratory for Structural Bioinformatics
U50: The upper 50-kDa domain of myosin
*V*_max_: Maximal velocity of the enzyme
*V*_max, ATP_: *V*_max_ for ATP
